# Consideration of body mass index (BMI) in the association between hand grip strength and hypertension: Korean Longitudinal Study of Ageing (KLoSA)

**DOI:** 10.1371/journal.pone.0241360

**Published:** 2020-10-29

**Authors:** Doukyoung Chon, Jaeyong Shin, Jae-Hyun Kim

**Affiliations:** 1 Department of Preventive Medicine, Korea University School of Medicine, Seoul, Korea; 2 Department of Preventive Medicine, School of Medicine, Ajou University, Suwon, Gyeonggi-do, Korea; 3 Department of Health Administration, College of Health Science, Dankook University, Cheonan, Korea; Ehime University Graduate School of Medicine, JAPAN

## Abstract

**Objective:**

The purpose of this study was to investigate the association between grip strength and hypertension in the Korean population aged 65 years or older. Furthermore, individual differences in BMI were taken into account to examine whether grip strength or a relative grip strength predicted hypertension better.

**Methods:**

Data from the Korean Longitudinal Study of Ageing from 2006 to 2016 were assessed, and a total of 3,383 participants were analyzed in our study (Male: 1,527, Female: 1,856). Using the generalized estimating equation model, the association between grip strength and hypertension, assessed by the response to the question ‘have you ever been diagnosed with hypertension from your doctor?’, over the follow-up period was analyzed. The relative grip strength, calculated by dividing the mean grip strength by BMI, was also analyzed in association of hypertension.

**Results:**

Both grip strength and relative grip strength were significantly associated with hypertension in our sample. However, the results were more significant in the total sample when relative grip strength was used. In terms of grip strength, as the High group as reference: Low (Odds Ratio (OR): 1.238, 95% Confidence Interval (CI): 1.096, 1.397), Middle Low (OR: 1.104, 95% CI: 0.990, 1.231), and Middle high (OR: 1.024, 95% CI: 0.934, 1.122). In the analysis using relative grip strength, as High group as reference: Low (OR: 1.393, 95% CI: 1.234, 1.573), Middle low (OR: 1.232, 95% CI: 1.104, 1.374), and Middle high (OR:1.104, 95% CI: 1.009, 1.209). Furthermore, the lower QIC measure in the model with relative grip strength (QIC: 25,251) compared with the one using grip strength (QIC: 25,266) indicated a better model fit in the former.

**Conclusions:**

The results of the current study strengthen the previous findings in regards to hand grip strength and health. Furthermore, the results of our study shines light on the necessity of considering individual differences in BMI, when using a physical measure as a study variable.

## Introduction

Despite the advancement in medicine, mortality due to circulatory diseases remains high across the world [1], and this chronic condition can be affected by various lifestyle risk factors. Also, health problems accumulate as an individual ages, and subsequently, age is strongly related to the risk of such an illness [2]. Notably, hypertension, one example of circulatory diseases, can be regarded as a chronic condition on its own, but furthermore, it could lead to problems such as cardiovascular disease [3], hospitalization [4], as well as poor health-related quality of life [5]. Therefore, it is important to study the factors that could predict the risk of hypertension in order to implement behavioral interventions.

Hypertension is one of the major chronic conditions affecting the older population, and in 2017, approximately 60% of people 60 years and older were reported to having hypertension in Korea [6]. Furthermore, according to previous studies, hypertension can be aggravated by factors such as poor diet, obesity and weak muscle strength [7]. As such, an individual’s level of physical fitness is an important determinant in association with hypertension, and in previous studies, hand-grip strength (HGS) measurement has often been used as an inference to physical health [8,9].

HGS has been used as an efficient measure of musculoskeletal health in previous studies, and its usefulness has been documented in regards to hypertension as well [10]. HGS is being used in various epidemiological studies, because it is easy to administer and does not cause much harm to the subject of the study [11,12]. For instance, a negative association between grip strength and hypertension has been found in various studies [9,13]. However, not many studies have considered individual differences in body mass index (BMI) in relation to HGS, which should be included in analyses considering previous studies. For instance, Ji et al. [7] found that the association between HGS and hypertension differed depending on people’s physical stature. Furthermore, according to a meta-analytic study [14], BMI and HGS were significantly associated with one another, and this association need to be considered more.

Korea is a country with one of the fastest growing elderly population in the world [1], and because age is strongly associated with hypertension, it is reasonable to assume that the prevalence of hypertension will continue to rise. Therefore, it is important to come up with ways to better predict hypertension so that interventions to prevent or delay the onset of hypertension can be implemented to the at-risk population. Keeping the findings of previous studies in regard, we hypothesized that the level of HGS would be able to predict the onset of hypertension in the Korean elderly population, and that this predictability would improve when we consider the individual differences in BMI in our analysis.

## Materials and methods

### Data source & study sample

The data used for the following analyses were derived from the Korean Longitudinal Study of Ageing (KLoSA) in 2006, 2008, 2010, 2012, 2014 and 2016. KLoSA data was gathered for the purpose of preparing for the aged society in terms of system reform and policy decision. The data is composed of 7 categories such as population, family, health, employment, income, wealth, subjective expectation and life expectation. This biennial survey involves multistage stratified sampling based on geographical areas and housing types across Korea. Participants were selected randomly using a multistage, stratified probability sampling design to create a nationally representative sample of community-dwelling Koreans 45 years of age and older. Participant selection was performed by the Korea Labor Institute for these rapidly growing populations, including individuals from both urban and rural areas. In case of refusal to participate, another subject was selected from an additional, similar sample from the same district. From the original 10,254 participants, those aged 65 years and older were included in our analysis. In our final analysis, 3,383 participants (Male: 1,527, Female: 1,856) without missing values on the variables of interest (e.g., grip strength, BMI, heart disease, Mini Mental State Examination (MMSE)), were included. Out of the public data in Korea, KLoSA was considered as the most suitable data for the analysis involved in the current study.

### Dependent variables

#### The presence of circulatory related diseases

The dependent variable was the diagnosis of hypertension by a doctor. The participants were asked the following question, ‘Have you ever been diagnosed with hypertension from your doctor?’ The response to the question was dichotomized as either ‘yes’ or ‘no’. This response was collected from all waves so that the possible changes in the presence of hypertension can be accounted for in the final analysis.

### Independent variables

#### Grip strength and relative grip strength

The independent variable was grip strength. Grip strength was measured by a handgrip dynamometer (Model number: NO6103, Manufacturer: TANITA, Japan). The test was performed in a sitting position with the elbow flexed at 90° on both the right and the left sides. The mean strength was calculated from grip strengths on both sides [15]. Grip strength in each year was divided into four groups: Q1, Q2, Q3, Q4 using SAS Rank function. For relative grip strength, grip strength was divided by BMI, which was calculated from the reported height and weight (kg/m^2^). Grip strength at all waves were used in the final analysis to account for the possible changes in the strength of participants.

#### Control variables

This study used educational level (elementary school or less, middle school, high school, and college or more), gender (male or female), age (65–69 years old, 70–74, and 85 years or more)residential region (metropolitan (e.g. Seoul), urban (e.g. Daejeon, Daegu, Busan, Incheon, Kwangju, or Ulsan), or rural (not classified as administrative of a city), national health insurance (health insurance, medical aid), Mini Mental State Examination (MMSE) (dementia (0–17), cognitive decline (18–23), normal (24–30)), smoking status (smoker, former smoker, never), alcohol use (never, former drinker, drinker), labor (yes or no), BMI (thin (0–18.4), moderate (18.5–22.9), overweight (23–24.9), obese (>25)), heart disease (yes or no), Year (2006, 2008, 2010, 2012, 2014, 2016) as covariates.

#### Analytical approach and statistics

Chi-square test, and generalized estimating equation (GEE) regression model with a binary distribution which controls for characteristics that change over time, such as confounding variables, were used to investigate the association between degree of grip strength and hypertension. The GEE model is a useful analytical tool for longitudinal studies, because it offers a way to handle unbalanced and missing data. For example, the GEE is able to control for the change in the presence of hypertension over time. In GEE, *proc genmod* was used, with *link logit*, *distribution normal*. For all analyses, SAS statistical software package, version 9.4 (SAS Institute, Inc., Cary, NC, USA) was used. All statistical tests were two-tailed, with the null hypothesis of no difference being rejected if p < 0.05.

## Results

### General characteristics

Table 1 shows the general characteristics of the participants. The participants had a mean age of 73.354 (Standard Deviation (SD): 6.217), mean grip strength of 21.934 (SD: 7.768), mean BMI of 23.114 (SD: 6.779), and mean relative grip strength of 0.968 (SD: 0.356). Of the 3,383 participants selected for the study, 1,335 (39.5%) people had hypertension, and both grip strength and relative grip strength showed a significant chi-square value (*p*-value: < .001). In terms of grip strength, people with hypertension in the Low group, ranging between 1.250 and 16.475, were 391 (45.1%), Middle low, ranging between 16.50 and 21.00, 382 (41.7%), Middle high, ranging between 21.025 and 28.225, 280 (36.9%) and High, ranging between 28.250 and 84.300, 282 (33.6%). In terms of relative grip strength, people in the Low group were 419 (47.5%), Middle low 376 (45.1%), Middle high 305 (35.8%) and 235 (28.8%) in the High relative grip strength group. More female participants (N: 808) than male participants (N: 527) experienced hypertension, and there were significant differences in hypertension (*p*-value: < .001) between age groups. In terms of other control variables, national health insurance status (*p*-value: .015), smoking status (*p*-value: < .001), labor status (*p*-value: < .001), and the presence of heart disease (*p*-value: < .001) differed significantly in terms of hypertension, but the rest did not.

**Table 1:** General characteristics of subjects included for analysis.

			Hypertension				P-value
	Total		Yes		No		
	N	%	N	%	N	%	
**Grip strength**							< .0001
**Low**	868	25.7	391	45.1	477	55.0	
**Middle Low**	916	27.1	382	41.7	534	58.3	
**Middle High**	759	22.4	280	36.9	479	63.1	
**High**	840	24.8	282	33.6	558	66.4	
**Relative Grip Strength**							< .0001
**Low**	882	26.1	419	47.5	463	52.5	
**Middle Low**	833	24.6	376	45.1	457	54.9	
**Middle High**	851	25.2	305	35.8	546	64.2	
**High**	817	24.2	235	28.8	582	71.2	
**Education level**							0.243
**≤ Elementary school**	2,339	69.1	905	38.7	1,434	61.3	
**Middle school**	368	10.9	157	42.7	211	57.3	
**High school**	470	13.9	182	38.7	288	61.3	
**≥ College**	206	6.1	91	44.2	115	55.8	
**Gender**							< .0001
**Male**	1,527	45.1	527	34.5	1,000	65.5	
**Female**	1,856	54.9	808	43.5	1,048	56.5	
**Age**							0.004
**65–69**	1,343	39.7	486	36.2	857	63.8	
**70–74**	990	29.3	400	40.4	590	59.6	
**≥85**	1,050	31.0	449	42.8	601	57.2	
**Residential region**							0.275
**Metropolitan**	600	17.7	254	42.3	346	57.7	
**Urban**	870	25.7	341	39.2	529	60.8	
**Rural**	1,913	56.6	740	38.7	1,173	61.3	
**National health insurance**							0.015
**Health insurance**	3,109	91.9	1,208	38.9	1,901	61.2	
**Medical aid**	274	8.1	127	46.4	147	53.7	
**MMSE**							0.813
**Dementia**	464	13.7	184	39.7	280	60.3	
**Cognitive decline**	853	25.2	344	40.3	509	59.7	
**Normal**	2,066	61.1	807	39.1	1,259	60.9	
**Smoking status**							< .0001
**Smoker**	2,428	71.8	1,031	42.5	1,397	57.5	
**Former smoker**	408	12.1	151	37.0	257	63.0	
**Never**	547	16.2	153	28.0	394	72.0	
**Alcohol use**							0.726
**Never**	3,071	90.8	1,209	39.4	1,862	60.6	
**Former Drinker**	312	9.2	126	40.4	186	59.6	
**Drinker**	0	0.0	0	0.0	0	0.0	
**Labor**							< .0001
**Yes**	642	19.0	197	30.7	445	69.3	
**No**	2,741	81.0	1,138	41.5	1,603	58.5	
**BMI**							< .0001
**Thin**	220	6.5	51	23.2	169	76.8	
**Moderate**	2,023	59.8	700	34.6	1,323	65.4	
**Overweight**	848	25.1	409	48.2	439	51.8	
**Obese**	292	8.6	175	59.9	117	40.1	
**Heart disease**							< .0001
**Yes**	254	7.5	149	58.7	105	41.3	
**No**	3,129	92.5	1,186	37.9	1,943	62.1	

*MMSE: Mini-Mental State Examination.

*Grip strength values: Low-1.250~16.475, Middle Low-16.50~21.00, Middle High-21.025~28.225, High-28.250~84.300.

*Relative grip strength values: Low-0.000~0.709, Middle Low-0.709~0.925, Middle High-0.925~1.239, High-1.239~4.303.

### Adjusted association between grip strength and hypertension

The results of the fully adjusted model are shown in Table 2. In the total sample, the association between grip strength and hypertension was only statistically significant in the Low group (OR: 1.238, 95% CI: 1.096, 1.397) as the High group as reference. However, the results showed slightly different trends when gender was taken into account. The results did not differ much in the male sample, but in terms of the female sample, the associations between grip strength and hypertension were statistically significant in all grip strength groups: Low (OR: 1.684, 95% CI: 1.252, 2.265), Middle low (OR: 1.584, 95% CI: 1.180, 2.126), and Middle high (OR 1.482, 95% CI: 1.099, 1.999) with High as reference. Furthermore, there was a gradient increase in the risk of hypertension in accordance with weaker grip strength, providing indication to the importance of taking gender into account.

**Table 2:** Adjusted effect of grip strength on hypertension.

	Hypertension											
	Total				Male				Female			
	OR	95% CI		P-value	OR	95% CI		P-value	OR	95% CI		P-value
**Grip strength**												
**Low**	1.238	1.096	1.397	0.001	1.446	1.155	1.812	0.001	1.684	1.252	2.265	0.001
**Middle Low**	1.104	0.990	1.231	0.076	1.069	0.911	1.255	0.413	1.584	1.180	2.126	0.002
**Middle High**	1.024	0.934	1.122	0.618	1.035	0.932	1.150	0.515	1.482	1.099	1.999	0.010
**High**	1.000				1.000				1.000			
**Education level**												
**≤ Elementary school**	0.894	0.786	1.017	0.088	0.852	0.733	0.989	0.035	1.021	0.761	1.369	0.893
**Middle school**	1.114	0.968	1.282	0.132	1.024	0.868	1.208	0.777	1.382	1.013	1.885	0.041
**High school**	1.124	0.981	1.287	0.091	1.228	1.054	1.430	0.008	0.958	0.698	1.316	0.792
**≥ College**	1.000				1.000				1.000			
**Gender**												
**Male**	0.880	0.795	0.975	0.014								
**Female**	1.000											
**Age**												
**65–69**	1.000				1.000				1.000			
**70–74**	1.268	1.175	1.369	< .0001	1.219	1.087	1.367	0.001	1.307	1.179	1.450	< .0001
**≥85**	1.601	1.479	1.733	< .0001	1.490	1.323	1.679	< .0001	1.700	1.527	1.892	< .0001
**Residential region**												
**Metropolitan**	1.125	1.032	1.227	0.007	1.104	0.971	1.256	0.132	1.158	1.030	1.301	0.014
**Urban**	0.953	0.888	1.023	0.183	0.899	0.807	1.000	0.051	1.004	0.913	1.103	0.942
**Rural**	1.000				1.000				1.000			
**National health insurance**												
**Health insurance**	0.740	0.656	0.834	< .0001	0.637	0.526	0.771	< .0001	0.823	0.706	0.960	0.013
**Medical aid**	1.000				1.000				1.000			
**MMSE**												
**Dementia**	1.166	1.052	1.292	0.004	1.110	0.901	1.368	0.328	1.162	1.029	1.312	0.015
**Cognitive decline**	1.062	0.986	1.143	0.113	1.004	0.891	1.131	0.946	1.088	0.989	1.197	0.082
**Normal**	1.000				1.000				1.000			
**Smoking status**												
**Smoker**	1.421	1.279	1.579	< .0001	1.496	1.329	1.684	< .0001	1.145	0.889	1.473	0.295
**Former smoker**	1.369	1.224	1.532	< .0001	1.398	1.240	1.575	< .0001	1.121	0.761	1.652	0.564
**Never**	1.000				1.000				1.000			
**Alcohol use**												
**Never**	1.000				1.000				1.000			
**Former Drinker**	1.077	0.919	1.263	0.359	1.348	1.020	1.782	0.036	1.026	0.782	1.345	0.854
**Drinker**	1.267	1.082	1.484	0.003	1.519	1.155	1.998	0.003	1.368	1.053	1.777	0.019
**Labor**												
**Yes**	0.795	0.738	0.856	< .0001	0.776	0.702	0.858	< .0001	0.794	0.711	0.887	< .0001
**No**	1.000				1.000				1.000			
**BMI**												
**Thin**	1.000				1.000				1.000			
**Moderate**	1.882	1.629	2.175	< .0001	2.115	1.680	2.664	< .0001	1.728	1.431	2.086	< .0001
**Overweight**	3.219	2.766	3.747	< .0001	3.784	2.972	4.819	< .0001	2.827	2.319	3.445	< .0001
**Obese**	5.318	4.474	6.321	< .0001	6.720	5.047	8.949	< .0001	4.557	3.658	5.676	< .0001
**Heart disease**												
**Yes**	2.027	1.829	2.246	< .0001	2.058	1.762	2.404	< .0001	1.981	1.727	2.273	< .0001
**No**	1.000				1.000				1.000			
**Year**												
**2006**	0.686	0.619	0.761	< .0001	0.661	0.565	0.773	< .0001	0.691	0.601	0.794	< .0001
**2008**	0.807	0.728	0.894	< .0001	0.793	0.679	0.927	0.004	0.805	0.701	0.925	0.002
**2010**	0.949	0.856	1.053	0.326	0.932	0.798	1.089	0.375	0.955	0.830	1.099	0.518
**2012**	1.000	0.903	1.107	0.996	1.003	0.861	1.168	0.974	0.989	0.862	1.135	0.876
**2014**	1.055	0.923	1.207	0.431	1.024	0.867	1.208	0.784	1.081	0.835	1.400	0.555
**2016**	1.000				1.000				1.000			
**QIC**	25,266				11,227				14,075			

* BMI–body mass index.

* MMSE–mini-mental state examination.

* QIC–quasi-information criterion.

### Adjusted association between relative grip strength and hypertension

Considering relative grip strength in association with hypertension (Table 3), the results were as follows: Low (OR: 1.393, 95% CI: 1.234, 1.573), Middle low (OR: 1.232, 95% CI: 1.104, 1.374), and Middle High (OR: 1.104, 95% CI: 1.009, 1.209) with High as reference. When relative grip strength was used, the OR for all categories of grip strength were statistically significant and this was also true in the case of males. Contrarily, relative grip strength was only statistically significantly associated with hypertension for females in the Low group (OR: 1.356, 95%: 1.356, 1.777). Furthermore, the Quasi-information criterion (QIC) measures for the total sample as well as gender stratified samples using relative grip strength were lower than that using grip strength, indicating a better model fit with the use of relative grip strength as a measure of physical fitness. We compared the difference between the relative grip strength and grip strength further by creating the receiver operating characteristic (ROC) curves for both. The bigger area under the curve (AUC) in the case of using the relative grip strength in association with hypertension provides additional support for considering BMI in the association between HGS and hypertension.

**Table 3:** Adjusted effect of relative grip strength on hypertension.

	Hypertension				Hypertension				Hypertension			
	Total				Male				Female			
	OR	95% CI		P-value	OR	95% CI		P-value	OR	95% CI		P-value
**Relative Grip strength**												
**Low**	1.393	1.234	1.573	< .0001	1.397	1.107	1.763	0.005	1.356	1.035	1.777	0.027
**Middle Low**	1.232	1.104	1.374	0.000	1.178	1.003	1.382	0.046	1.213	0.929	1.584	0.156
**Middle High**	1.104	1.009	1.209	0.032	1.148	1.035	1.272	0.009	1.020	0.777	1.340	0.887
**High**	1.000				1.000				1.000			
**Education level**												
**≤ Elementary school**	0.887	0.780	1.009	0.068	0.850	0.732	0.987	0.033	1.022	0.761	1.371	0.887
**Middle school**	1.107	0.962	1.274	0.157	1.019	0.864	1.203	0.819	1.392	1.021	1.900	0.037
**High school**	1.123	0.980	1.286	0.095	1.227	1.054	1.430	0.009	0.965	0.703	1.325	0.824
**≥ College**	1.000				1.000				1.000			
**Gender**												
**Male**	0.930	0.841	1.028	0.157								
**Female**	1.000											
**Age**												
**65–69**	1.000				1.000				1.000			
**70–74**	1.264	1.171	1.365	< .0001	1.208	1.077	1.355	0.001	1.299	1.172	1.441	< .0001
**≥85**	1.590	1.470	1.720	< .0001	1.463	1.300	1.647	< .0001	1.681	1.512	1.869	< .0001
**Residential region**												
**Metropolitan**	1.120	1.027	1.221	0.010	1.098	0.966	1.250	0.154	1.153	1.026	1.296	0.017
**Urban**	0.953	0.888	1.023	0.182	0.898	0.807	1.000	0.050	1.000	0.910	1.099	0.998
**Rural**	1.000				1.000				1.000			
**National health insurance**												
**Health insurance**	0.741	0.658	0.835	< .0001	0.638	0.527	0.772	< .0001	0.830	0.712	0.967	0.017
**Medical aid**	1.000				1.000				1.000			
**MMSE**												
**Dementia**	1.154	1.042	1.279	0.006	1.110	0.901	1.367	0.326	1.131	1.002	1.277	0.046
**Cognitive decline**	1.055	0.980	1.136	0.157	0.999	0.887	1.125	0.980	1.076	0.978	1.183	0.133
**Normal**	1.000				1.000				1.000			
**Smoking status**												
**Smoker**	1.419	1.277	1.577	< .0001	1.496	1.328	1.684	< .0001	1.139	0.885	1.467	0.312
**Former smoker**	1.372	1.226	1.534	< .0001	1.401	1.243	1.578	< .0001	1.118	0.759	1.648	0.573
**Never**	1.000				1.000				1.000			
**Alcohol use**												
**Never**	1.000				1.000				1.000			
**Former Drinker**	1.079	0.921	1.265	0.347	1.342	1.015	1.774	0.039	1.022	0.779	1.340	0.874
**Drinker**	1.265	1.080	1.482	0.004	1.510	1.148	1.986	0.003	1.365	1.050	1.773	0.020
**Labor**												
**Yes**	0.802	0.745	0.864	< .0001	0.780	0.705	0.862	< .0001	0.804	0.720	0.899	0.000
**No**	1.000				1.000				1.000			
**BMI**												
**Thin**	1.000				1.000				1.000			
**Moderate**	1.785	1.544	2.063	< .0001	2.017	1.603	2.537	< .0001	1.629	1.348	1.969	< .0001
**Overweight**	2.940	2.523	3.425	< .0001	3.486	2.738	4.438	< .0001	2.536	2.075	3.099	< .0001
**Obese**	4.677	3.924	5.576	< .0001	5.930	4.444	7.913	< .0001	3.939	3.145	4.933	< .0001
**Heart disease**												
**Yes**	2.021	1.824	2.239	< .0001	2.049	1.754	2.394	< .0001	1.969	1.716	2.259	< .0001
**No**	1.000				1.000				1.000			
**Year**												
**2006**	0.682	0.615	0.756	< .0001	0.653	0.558	0.764	< .0001	0.692	0.602	0.795	< .0001
**2008**	0.801	0.723	0.888	< .0001	0.782	0.669	0.914	0.002	0.806	0.702	0.925	0.002
**2010**	0.940	0.848	1.043	0.245	0.922	0.789	1.076	0.304	0.951	0.827	1.094	0.483
**2012**	0.993	0.897	1.100	0.897	0.993	0.853	1.157	0.929	0.988	0.861	1.134	0.864
**2014**	1.059	0.926	1.211	0.401	1.024	0.867	1.209	0.779	1.084	0.837	1.404	0.540
**2016**	1.000				1.000				1.000			
**QIC**	25,251				11,225				14,066			

* BMI–body mass index.

* MMSE–mini-mental state examination.

* QIC–quasi-information criterion.

## Discussion

In this study, we were able to utilize a nationally representative data of Korea to find better ways to predict hypertension in the elderly population. Similar to previous studies [13,16], the results from our study indicated a significant association between HGS and hypertension. Moreover, considering previous studies [17,18], which presented significant association between relative grip strength and biomarkers (e.g., blood pressure), we included BMI in this association and performed a separate analysis using a relative grip strength. The results of our study presented a more significant association between relative grip strength and hypertension compared with grip strength and hypertension. Also, using relative grip strength in the analysis improved the model fit of the analysis compared with when grip strength was used. Although studies considering BMI in the association between HGS and hypertension are scarce, studies in this field of research have provided evidence to the importance of considering body composition [19,20]. Therefore, it is plausible to use the relative grip strength in future studies, and interpret the results accordingly.

In the past, Gale et al. [21] included BMI and HGS as separate variables in association with cardiovascular mortality, but contrary to our study, BMI remained significant in women, and not in men. Given the fact that the outcomes of the two studies as well as the method, in which, BMI was used as a variable differed, it is worth noting the differing effect of BMI between genders in similar circumstances. Also, the QIC measure provided in our study strengthens the plausibility of our model including BMI. Furthermore, Dong et al. [22] found that higher HGS was associated with low blood pressure, but an inverse association when BMI was included in the analysis. The discrepancy between their study findings and ours may be due to the age difference in sample groups. Whereas the participants in our study were older adults 65 years and above, the participants in Dong et al.’s study were teenagers. Accordingly, comparing varying age groups in regards to the association between BMI adjusted HGS and hypertension could provide valuable information for better predictive ability.

There are a few possible mechanisms relating HGS to hypertension. First, decreased physical activity has shown to be associated with hypertension [23], and that people with poor strength reported more difficulty exercising [24]. Because HGS is a widely recommended measure of muscle strength [25], it is possible to consider that people with low HGS are less physically **active**, consequently increasing their risk of hypertension. Therefore, HGS could be used as a useful predictive indicator of hypertension in a clinical setting, as well as a reason to prescribe more exercise to those who exhibit low HGS. Another mechanism that could associate HGS to hypertension is the arterial structure. A previous study reported an improved structure in the brachial artery due to isometric handgrip exercise [26]. Accordingly, lower HGS could be associated with poor status of the brachial artery, which in term could lead to hypertension. Subsequently, a type of exercise training could be recommended to those with hypertension, as well as those at-risk of obtaining hypertension.

The findings of this study provide important implications for the elderly population of Korea, as well as present various strengths. First, the data used in this study were based on a nationally representative sample of 65 years and older. Therefore, the generalizability of the study is viable. Also, the residential regions of the study subjects were controlled for in our analysis in order to reduce the possibly biased results from over sampling in a certain region of Korea. Second, this was a prospective cohort data with 10 years of follow-up and a good follow-up rate (78.8%). This was important for our analysis, because we only included a specific age group of the entire KLoSA sample and the ones with no missing values for the variables of interest. Acquiring a good number of final sample enabled us to reach a good statistical power to show valuable findings. Furthermore, the longitudinal design of our analysis enabled us to infer a causal relationship between HGS and hypertension.

Despite the strengths of our study, the limitations need to be considered as well. Even though the results are generalizable to the Korean elderly population, it is unable to represent more specific populations, such as people who are hospitalized. Therefore, research in this line of work should continue considering more variety of population. Also, regardless of the good follow-up rate, there were people who dropped out of the study, and could have biased our results. Furthermore, a possibility of selection bias due to differences in characteristics between included and excluded participants, and a misclassification bias due participants falsely reporting the presence of hypertension, need to be taken into account. Lastly, the height and the weight variables were based on reported values instead of physical measurements, possibly reducing the accuracy of the BMI value used in our study. In the future, considering more various age groups will provide additional information regarding HGS and hypertension. Also, researchers and clinicians should try to develop interventions to prevent hypertension, as well as ameliorate the severity of those suffering from it.

## References

[1] Indicators OJOI, OECD Publishing, Paris https://doiorg/101787/health_glance—en Accessed February. Health at a Glance 2011. 2015; 15:2016.

[2] Heidenreich PA, Trogdon JG, Khavjou OA, Butler J, Dracup K, Ezekowitz MD, et al Forecasting the future of cardiovascular disease in the United States: a policy statement from the American Heart Association. Circulation 2011; 123:933–944. 10.1161/CIR.0b013e31820a55f5 21262990

[3] Wall HK, Hannan JA, Wright JS. Patients with undiagnosed hypertension: hiding in plain sight. JAMA 2014; 312:1973–1974. 10.1001/jama.2014.15388 25399269 PMC4596255

[4] Liu L, An Y, Chen M, Liu Z, Hu X, Chou E, et al Trends in the prevalence of hospitalization attributable to hypertensive diseases among United States adults aged 35 and older from 1980 to 2007. The American Journal of Cardiology 2013; 112:694–699. 10.1016/j.amjcard.2013.04.050 23726180

[5] Alonso J, Ferrer M, Gandek B, Ware JE, Aaronson NK, Mosconi P, et al Health-related quality of life associated with chronic conditions in eight countries: results from the International Quality of Life Assessment (IQOLA) Project. Springer 2004; 13:283–298. 10.1023/b:qure.0000018472.46236.05 15085901

[6] Korea S. Population Prevalence of Chronic Conditions. Statistics Korea. 2019.

[7] Ji C, Zheng L, Zhang R, Wu Q, Zhao Y. Handgrip strength is positively related to blood pressure and hypertension risk: results from the National Health and nutrition examination survey. Lipids in Health and Disease 2018; 17:86 10.1186/s12944-018-0734-4 29665844 PMC5904981

[8] Mainous AG III, Tanner RJ, Anton SD, Jo A. Grip strength as a marker of hypertension and diabetes in healthy weight adults. American Journal of Preventive Medicine 2015; 49:850–858. 26232901 10.1016/j.amepre.2015.05.025 PMC4656117

[9] Lee J-A. Relationship between Grip Strength and Prevalence of Hypertension in Korean Adults: the Sixth Korea National Health and Nutrition Examination Survey (2015). Official Journal of the Korean Academy of Kinesiology 2017; 19:53–60.

[10] Gubelmann C, Vollenweider P, Marques-Vidal P. Association of grip strength with cardiovascular risk markers. European Journal of Preventive Cardiology 2017; 24:514–521. 10.1177/2047487316680695 27885059

[11] Silventoinen K, Magnusson PK, Tynelius P, Batty GD, Rasmussen F. Association of body size and muscle strength with incidence of coronary heart disease and cerebrovascular diseases: a population-based cohort study of one million Swedish men. International Journal of Epidemiology 2008; 38:110–118. 10.1093/ije/dyn231 19033357

[12] Artero EG, Lee D-C, Lavie CJ, España-Romero V, Sui X, Church TS, et al Effects of muscular strength on cardiovascular risk factors and prognosis. Journal of Cardiopulmonary Rehabilitation and Prevention 2012; 32:351 10.1097/HCR.0b013e3182642688 22885613 PMC3496010

[13] Leong DP, Teo KK, Rangarajan S, Lopez-Jaramillo P, Avezum A Jr, Orlandini A, et al Prognostic value of grip strength: findings from the Prospective Urban Rural Epidemiology (PURE) study. Lancet 2015; 386:266–273. 10.1016/S0140-6736(14)62000-6 25982160

[14] Hardy R, Cooper R, Sayer AA, Ben-Shlomo Y, Cooper C, Deary IJ, et al Body mass index, muscle strength and physical performance in older adults from eight cohort studies: the HALCyon programme. PLoS ONE 2013; 8:e56483 10.1371/journal.pone.0056483 23437142 PMC3577921

[15] Min J-Y, Lee K-J, Park J-B, Cho S-I, Park S-G, Min K. Social engagement, health, and changes in occupational status: analysis of the Korean Longitudinal Study of Ageing (KLoSA). PLoS ONE 2012; 7:e46500 10.1371/journal.pone.0046500 23056323 PMC3462751

[16] Rantanen T, Volpato S, Luigi Ferrucci M, Eino Heikkinen M, Fried LP, Guralnik JM. Handgrip strength and cause‐specific and total mortality in older disabled women: exploring the mechanism. The American Geriatrics Society 2003; 51:636–641. 10.1034/j.1600-0579.2003.00207.x 12752838

[17] Lee W-J, Peng L-N, Chiou S-T, Chen L-K. Relative handgrip strength is a simple indicator of cardiometabolic risk among middle-aged and older people: a nationwide population-based study in Taiwan. PLoS ONE 2016; 11:e0160876 10.1371/journal.pone.0160876 27559733 PMC4999244

[18] Lawman HG, Troiano RP, Perna FM, Wang C-Y, Fryar CD, Ogden CL. Associations of relative handgrip strength and cardiovascular disease biomarkers in US adults, 2011–2012. Amerian Journal of Preventive Medicine 2016; 50:677–683. 10.1016/j.amepre.2015.10.022 PMC7337414 26689977

[19] Fowles J, Roy J, Clarke J, Dogra S. Are the fittest Canadian adults the healthiest. 2014; 25:13–18. 24850392

[20] ChoquetteS, BouchardD, DoyonC, SénéchalM, BrochuM, DionneIJ. Relative strength as a determinant of mobility in elders 67–84 years of age. A nuage study: Nutrition as a determinant of successful aging. The Journal of Nutrition Health and Aging 2010; 14:190–195. 10.1007/s12603-010-0047-4 20191251

[21] Gale CR, Martyn CN, Cooper C, Sayer AA. Grip strength, body composition, and mortality. International Journal of Epidemiology 2006; 36:228–235. 10.1093/ije/dyl224 17056604

[22] Dong B, Wang Z, Arnold L, Song Y, Wang H-J, Ma J. The association between blood pressure and grip strength in adolescents: does body mass index matter? Hypertension Research 2016; 39:919 10.1038/hr.2016.84 27383511

[23] Sharman JE, Stowasser M. Australian association for exercise and sports science position statement on exercise and hypertension. Journal of Science and Medicine in Sport 2009; 12:252–257. 10.1016/j.jsams.2008.10.009 19147407

[24] Rantanen T, Guralnik JM, Sakari-Rantala R, Leveille S, Simonsick EM, Ling S, et al Disability, physical activity, and muscle strength in older women: the Women's Health and Aging Study. Archives of Physical Medicine and Rehabilitation 1999; 80:130–135. 10025485 10.1016/s0003-9993(99)90109-0

[25] Roberts HC, Denison HJ, Martin HJ, Patel HP, Syddall H, Cooper C, et al A review of the measurement of grip strength in clinical and epidemiological studies: towards a standardised approach. Age and Ageing 2011; 40:423–429. 10.1093/ageing/afr051 21624928

[26] McGowan CL, Levy AS, Millar PJ, Guzman JC, Morillo CA, McCartney N, et al Acute vascular responses to isometric handgrip exercise and effects of training in persons medicated for hypertension. American Journal of Physiology Heart and Circulatory Physiology 2006; 291:H1797–H1802. 10.1152/ajpheart.01113.2005 16648182

