# Correction: Consideration of body mass index (BMI) in the association between hand grip strength and hypertension: Korean Longitudinal Study of Ageing (KLoSA)

**DOI:** 10.1371/journal.pone.0303220

**Published:** 2024-05-01

**Authors:** The PLOS ONE Editors

There are errors in the Author Contributions of the second author, Jaeyong Shin. The correct contributions are: Conceptualization, Data curation, Methodology, Supervision, Validation, Visualization, Writing—review & editing.

## References

[1] Chon D, Shin J, Kim J-H (2020) Consideration of body mass index (BMI) in the association between hand grip strength and hypertension: Korean Longitudinal Study of Ageing (KLoSA). PLoS ONE 15(10): e0241360. doi: 10.1371/journal.pone.0241360 33119673 PMC7595330

